# A Microwave-Assisted, Rapidly Self-Healing, FFF-Printed TPU and Its Application in Supercritical Foaming

**DOI:** 10.3390/nano16060384

**Published:** 2026-03-23

**Authors:** Shaoyun Chen, Rui Wang, Longhui Zheng, Jianhong Gao, Cuifang Cai, Zixiang Weng, Xiaoying Liu, Bo Qu, Jianlei Wang, Dongxian Zhuo

**Affiliations:** 1 School of Chemical Engineering and Materials Science, Quanzhou Normal University, Quanzhou 360002, China; 2 Fujian University Engineering Research Center of Polymer Functional Coating Based Graphene, Quanzhou 360002, China; 3 Fujian Key Laboratory of New Materials for Light Textile and Chemical Industry, Quanzhou 360002, China; 4 Fujian Key Laboratory of Nanomaterials, Fujian Institute of Research on the Structure of Matter, Chinese Academy of Sciences, Fuzhou 350002, China

**Keywords:** TPU, helical carbon nanotubes, supercritical foaming

## Abstract

To mitigate the interlayer defects and weak interfacial adhesion inherent in FFF-printed parts, thereby facilitating subsequent supercritical foaming applications, a microwave-assisted interlayer healing strategy is developed for FFF-printed, supercritical CO_2_-foamed thermoplastic polyurethane (TPU) by incorporating aminated helical multi-walled carbon nanotubes (AS-MWCNTs). Owing to their unique helical morphology, AS-MWCNTs exhibit enhanced microwave absorption and localized heating capability, enabling selective thermal activation at interlayer regions within the foamed architecture. Microwave irradiation induces localized softening of the TPU matrix and promotes polymer chain mobility and interdiffusion across layer interfaces, while preserving the cellular morphology and bulk foamed structure. By optimizing AS-MWCNT loading, substantial improvements in interlayer bonding strength, energy absorption, and overall mechanical performance are achieved. This work provides an effective strategy to restore interlayer integrity in supercritical CO_2_-foamed, additive manufactured elastomers and offers insights into the design of microwave-responsive, self-healing cellular materials.

## 1. Introduction

Thermoplastic polyurethane (TPU) is a high-performance segmented copolymer that synergistically combines elastomeric flexibility with thermoplastic processability [1,2]. Its microphase-separated structure, consisting of hard segments formed by urethane linkages and soft segments derived from long-chain diols, enables tunable mechanical properties across a wide hardness range, while maintaining exceptional elasticity, abrasion resistance, and low-temperature performance [3]. TPU can be repeatedly melted and reshaped using conventional thermoplastic techniques—including injection molding, extrusion, and calendaring—without chemical crosslinking, making it ideal for applications in footwear, medical devices, automotive components, and flexible electronics [4,5,6,7].

The development of 3D printing technology has brought revolutionary breakthroughs to footwear material design, enabling personalized customization and structural innovation beyond the reach of traditional manufacturing through precise digital modeling and layered fabrication. In the athletic footwear sector, technologies such as fused filament fabrication (FFF) and vat photopolymerization are employed to create lightweight, high-resilience midsole structures, thereby enhancing shock absorption performance and advancing sustainable footwear production while redefining the performance boundaries of footwear products [8,9]. Additive manufacturing of TPU has gained significant attention due to its flexibility, durability, and compatibility with FFF techniques [10,11].

Nowadays, supercritical foaming technology has gradually become a critical method for TPU foaming as it enables rapid foaming of TPU to form a low-density closed-cell structure, imparts excellent rebound performance, and achieves zero volatile organic compound (VOC) emissions during the molding process [12]. However, polymer structures fabricated via FFF modeling are highly susceptible to defects and poor interlayer adhesion due to insufficient polymer chain diffusion during layer-by-layer deposition and inadequate interlayer thermal bonding. This phenomenon not only compromises the mechanical, thermal, and electrical properties of FFF-printed polymer parts. It also renders the printed layers prone to delamination under the high-temperature and high-pressure conditions of subsequent supercritical foaming. Thereby impairing or even preventing successful foaming [13,14].

Recently, microwave-induced heating has emerged as a promising approach for non-contact, selective thermal treatment of polymeric materials. Thermoplastic polymers, such as TPU, inherently exhibit negligible microwave absorption, whereas conductive fillers demonstrate exceptional microwave absorption efficiency and undergo rapid localized thermal activation under microwave irradiation. Consequently, conductive filler-reinforced thermoplastic composites rapidly elevate in temperature under microwave exposure, leading to material softening and localized melting that effectively repairs internal microcracks [15,16,17]. In a wide range of fillers, carbon nanotubes (CNTs) are regarded as ideal candidates for polymer modification due to their excellent mechanical, electrical, and thermal properties [18,19]. CNTs also possess certain electromagnetic wave absorption capabilities and generate heat under irradiation, thereby being employed as welding materials for polymer layers [20,21]. Sweeney et al. achieved this by coating thermoplastic filaments with CNT-enriched polymer films and subsequent 3D printing, thereby forming a macroscopic architecture in which CNTs are selectively segregated at the interlayer interfaces. Upon microwave irradiation, these CNT-rich interfaces absorb electromagnetic energy, inducing localized heating, which enhances polymer chain mobility and entanglement within the weld zone. This mechanism enables controlled interfacial melting, facilitates molecular diffusion across layer boundaries, and ultimately results in significantly improved interlayer adhesion, reduced void formation, and enhanced mechanical integrity of the printed component [22]. Moreover, CNTs play a significant role in the supercritical foaming of thermoplastic polyurethane (TPU), effectively lowering the nucleation energy barrier during supercritical carbon dioxide foaming, significantly increasing foam density, and achieving a more uniform pore size distribution (<50 μm), thereby suppressing cell collapse and coalescence [23]. However, conventional CNTs exhibit relatively low microwave heating efficiency, limiting their applicability in microwave-assisted processing or in situ heating applications [24].

Helical multi-walled carbon nanotubes (S-MWCNTs) are a twisted, spiral-shaped morphology of CNTs [25,26]. The unique geometric structure of S-MWCNTs enhances electromagnetic coupling and dielectric loss, resulting in rapid and localized temperature elevation under microwave exposure. It also exhibits superior microwave absorption-heating efficiency compared to conventional straight-type CNTs [27,28,29]. However, applications of S-MWCNTs in microwave absorption-heating mechanisms within polymer matrices remain virtually unexplored.

Here, we composited amino-functionalized S-MWCNTS (AS-MWCNTs) with TPU, followed by FFF-printed modeling and microwave irradiation to fabricate S-MWCNTs/TPU FFF-printed parts with enhanced mechanical performance. This represents the first demonstration of microwave-induced rapid in situ heating of S-MWCNTs within a TPU matrix, enabling localized softening of printed layers, thereby improving interlayer adhesion and repairing interlayer defects to elevate the overall performance of TPU FFF-printed parts. The microwave-irradiated parts exhibit superior mechanical properties; under supercritical foaming conditions at 80 °C and a gas saturation time of 30 s, the resulting TPU FFF-printed foams display a uniform foam cell structure with excellent cell integrity. Importantly, this methodology is straightforward, enabling economically efficient, scalable production, and is particularly well-suited for applications in 3D-printed footwear and apparel.

## 2. Experiments

### 2.1. Materials

The polyester-based TPU elastomer TPU-65A, with a density of 1.16–1.18 g/cm^3^, was supplied by Wanhua Chemical Group Co., Ltd. (Yantai, China). S-MWCNTs (outer diameter: 100–200 nm, length: 1–10 μm) were provided by Beijing Boyu Gaoke New Material Co., Ltd. (Beijing, China). Polyethyleneimine (PEI, weight-average molecular weight: 1800 g/mol) was obtained from PolySciences, Inc. (Warrington, PA, USA). All other reagents were supplied by Shanghai Aladdin Biochemical Technology Co., Ltd. (Shanghai, China) and used without further purification.

### 2.2. Preparation of AS-MWCNTs

Prior to amination, the S-MWCNTs were first subjected to an oxidation treatment. Briefly, 3 g of S-MWCNTs were weighed and placed into a beaker. A mixture of concentrated sulfuric acid and concentrated nitric acid (volume ratio 3:1) was added. The beaker was then placed on a magnetic stirrer for agitation to facilitate heat dissipation and ensure thorough reaction, with magnetic stirring continued for 6 h. Subsequently, the stirred solution was sealed and allowed to stand for 1 day. The supernatant was decanted, and deionized water was added to bring the total volume to approximately 1 L. The mixture was then vacuum-filtered using a nylon membrane with a pore size of 0.22 μm, followed by repeated washing with water until the pH reached 7.

The amination process was conducted as follows: The oxidized S-MWCNTs prepared as above were placed in a beaker, and water was added to approximately 1 L for ultrasonic dispersion. The beaker was then transferred to an oil bath. Next, 1 g of PEI was added, and the mixture was magnetically stirred at a constant temperature of 60 °C for about 12 h to ensure complete reaction. After stirring, the solution was again subjected to vacuum filtration. The collected material was rinsed 3–4 times with acetone, followed by vacuum filtration and drying, yielding the AS-MWCNTs.

### 2.3. Preparation and FFF Printing of AS-MWCNTs/TPU Filaments

The quality of filaments and the parameter settings during printing are critical components for producing high-quality FFF parts. First, TPU pellets and AS-MWCNTs were dried in an oven at 80 °C for 2 h. The blended pellets were then fed into a HAAKE-PolyLab twin-screw extruder (MARS 60, Thermo Scientific, Waltham, MA, USA) for melt compounding and filament extrusion with a screw speed of 12 rpm, and the three heating zones of the extruder were set sequentially at 195 °C, 205 °C, and 210 °C. The produced filament was collected by a downstream winding machine. The obtained filament was pelletized, re-dried, and blended again, with this cycle repeated twice to ensure uniform dispersion of AS-MWCNTs within the TPU matrix. The prepared filaments contained AS-MWCNTs at 0, 1, 2, and 3 wt%, respectively, with the filament diameter controlled to 2.25 ± 0.05 mm. Samples were designed using Simplify software (3DGence SLICER 4), printing parameters were set (nozzle temperature: 215 °C, speed: 20 mm/s, layer thickness: 0.2 mm), and specimens were printed using a flexible F350 3D printer (Stratasys, Inc., Minneapolis, MN, USA).

### 2.4. Microwave Irradiation Treatment of AS-MWCNTs/TPU FFF-Printed Parts

The AS-MWCNTs/TPU parts fabricated via FFF printing, as described above, were subjected to microwave irradiation treatment under conditions of 2.45 GHz for an irradiation time of 12 s.

### 2.5. Supercritical Foaming of AS-MWCNTs/TPU FFF-Printed Parts

According to the preparation method reported in the previous literature [30], the microwave-treated FFF-printed parts were placed into a high-pressure autoclave connected to a CO_2_ gas supply. The parts were saturated in CO_2_ for 30 min at a saturation pressure of 5 MPa. Upon completion of the saturation step, the parts were rapidly transferred to an 80 °C hot water bath for a 30 s expansion process. Subsequently, the parts were removed and immersed in room-temperature water for 30 s to stabilize the foam structure. Finally, the foamed parts were taken out and dried in an oven to remove moisture. Analysis of the foamed parts was conducted 24 h after preparation to ensure structural stability prior to characterization. To produce AS-MWCNTs/TPU FFF-printed foams with uniform external dimensions, specific foaming conditions of 80 °C for 30 s were required.

### 2.6. Materials Characterization

The FT-IR spectra were obtained using a Thermo Scientific™ Nicolet™ iS10 Fourier transform infrared (FT-IR) spectrometer (Thermo Fisher Scientific, Waltham, MA, USA), furnished with an attenuated total reflectance (ATR) accessory. A total of 16 scans were collected at a resolution of 4 cm^−1^.

The melt flow index of the granular polymer samples was determined using an XNR-400H melt flow indexer (Beijing Zhonghang Times Instrument Equipment Co., Ltd., Beijing, China) with a 210 °C test temperature and 2.16 Kg load. It was calculated based on the collected material weight in 10 min.

Tensile properties were assessed utilizing an LD24 universal testing machine (Lishi (Shanghai) Scientific Instrument Co., Ltd., Shanghai, China), in compliance with ASTM D638-14 standards [31]. Tests were executed at a crosshead speed of 15 mm/min.

The structure of FFF-printed parts was scrutinized using a ZEISS Sigma 500 field emission scanning electron microscope (Carl Zeiss Microscopy GmbH, Oberkochen, Germany). This examination was conducted at an accelerating voltage of 5 kV, after the samples had been coated with gold to improve their conductivity.

## 3. Result and Discussion

### 3.1. FT-IR Characterization of AS-MWCNTs

**Figure 1:**
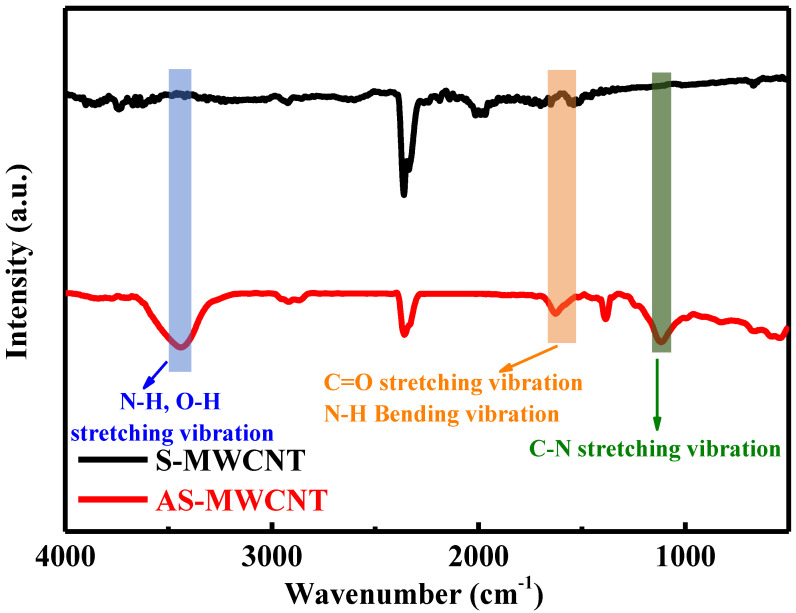
FT-IR spectra of S-MWCNT and AS-MWCNT.

The AS-MWCNTs were characterized by FT-IR spectroscopy before and after amination, with the results shown in Figure 1. The differences in the FT-IR spectra before and after amination were mainly attributed to the introduction of nitrogen-related functional groups and changes in the carbon skeleton vibration modes. In the spectrum of S-MWCNTs, the peak near 1580 cm^−1^ corresponded to the in-plane stretching vibration of the sp^2^-hybridized C=C bonds in the carbon skeleton [32]. After the amination treatment, nitrogen-containing groups such as carbonyl (-C=O), amino (-NH_2_), and imino (-NH-) were introduced onto the surface of the helical carbon nanotubes through chemical modification. New characteristic peaks appeared in the FT-IR spectrum: symmetric and asymmetric N-H stretching vibration doublets were observed in the 3300–3500 cm^−1^ region (primary amine -NH_2_ vibrations near 3350 cm^−1^ and 3450 cm^−1^; secondary amine –NH– vibration peaks in the 3300–3400 cm^−1^ range); C=O stretching vibration and N-H in-plane bending vibration peaks appeared between 1630 and 1550 cm^−1^ (C=O near 1620 cm^−1^, N-H near 1560 cm^−1^); and a weaker C-N stretching vibration peak was detected in the 1250–1020 cm^−1^ range [33]. These changes in the infrared characteristic peaks clearly demonstrate the modification of the surface chemical structure of the AS-MWCNTs by amination. This modification thereby creates favorable conditions for the effective integration of AS-MWCNTs with TPU.

### 3.2. Microstructural Analysis of AS-MWCNTs/TPU Composites

The microscopic morphology of the helical carbon nanotubes before and after amination was characterized by SEM, as shown in Figure S1. It can be observed that the amination treatment did not alter the microscopic morphology of the helical carbon nanotubes, indicating that no thick modified layer was formed on their surface. Furthermore, it is noteworthy that the amination modification process, due to the strong acid treatment, served to purify the helical carbon nanotubes. From the microscopic morphology of the AS-MWCNTs, a reduction in the surrounding impurity content was evident, a finding consistent with the results reported in the previous report [34].

### 3.3. Melt Flow Index (MFI) and Molding Shrinkage of AS-MWCNTs/TPU Composites

**Figure 2:**
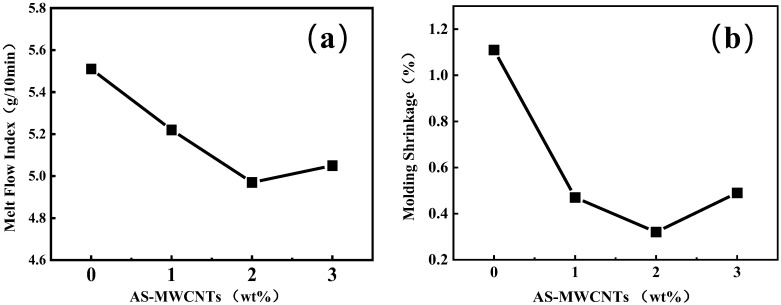
(**a**) Melt flow index of TPU and its composites. (**b**) Molding shrinkage of TPU and its composites.

The melt flow index (MFI) of the TPU composite incorporated with AS-MWCNTs was measured, and the results are presented in Figure 2. It can be observed that the AS-MWCNTs caused a continuous decrease in the MFI of TPU. This trend was fundamentally governed by the competitive mechanism between the interfacial interaction within the AS-MWCNTs/TPU matrix and the dynamic evolution of the filler network. At low loadings, -NH_2_ groups on AS-MWCNTs formed hydrogen bonds and dipole interactions with TPU’s urethane/ether segments, enhancing interfacial adhesion and restricting chain slippage. Simultaneously, AS-MWCNTs acted as nucleating agents, promoting hard-segment crystallization and further elevating melt resistance. As loading increased, a percolating network formed, maximizing physical cross-linking and severely limiting chain mobility, causing a sharp MFI drop. Beyond a critical threshold, agglomeration disrupted dispersion, creating rigid obstacles and stress concentrators [35].

TPU molding via FFF printing exhibited reduced molding shrinkage with AS-MWCNT addition, as shown in Figure 2b. At 1 wt%, amino groups formed hydrogen bonds with urethane and ether segments, restricting chain mobility and nucleating uniform hard-segment microcrystals, while the high modulus of AS-MWCNTs suppressed elastic recovery, collectively driving a sharp, near-linear shrinkage drop. At ~2 wt%, a percolating network saturated physical cross-linking, stabilizing chain constraint; microcrystal refinement continued, but crystallinity growth moderated due to increased chain immobilization, slowing shrinkage reduction. Simultaneously, the network absorbed thermal strain, minimizing interfacial debonding effects. At 3 wt%, agglomerates disrupted continuity, yet a high aspect ratio maintained partial constraint; elevated melt viscosity limited void contraction, balancing residual stress. Amination enabled earlier network saturation than pristine CNTs via stronger H-bonding, shifting the shrinkage transition point to lower loadings. Overall, AS-MWCNTs enabled precise shrinkage control for high-fidelity FFF printing.

### 3.4. Printability of AS-MWCNTs/TPU Composites via FFF

**Figure 3:**
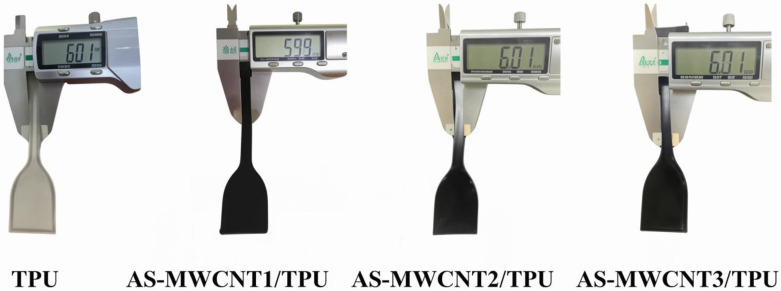
Dimensions of FFF-printed parts from TPU and its composites.

TPU and AS-MWCNTs/TPU composite filaments were used for FFF printing. The printed samples are shown in Figure 3. It was observed that the dimensions of the FFF-printed parts for both TPU and AS-MWCNTs/TPU composites deviated from the set printing dimension (6.00 mm) by only ±0.01 mm (±0.17%). This indicates that the incorporation of AS-MWCNTs does not adversely affect the FFF printability of TPU.

**Figure 4:**
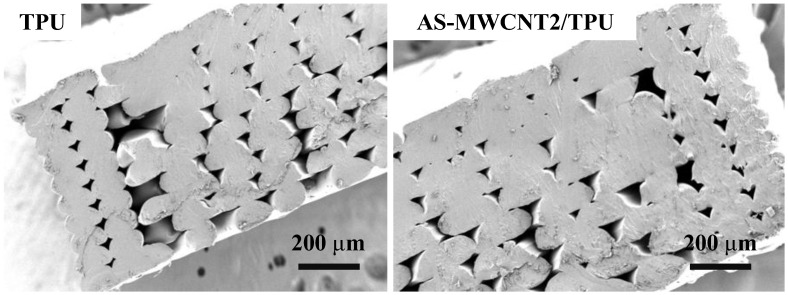
SEM images of cross-sections of FFF-printed TPU part and AS-MWCNT2/TPU part.

The cross-sections of the FFF-printed parts were analyzed by SEM, as shown in Figure 4. TPU and the AS-MWCNT2/TPU composite exhibited similar cross-sections of the FFF-printed parts. This further demonstrates that the addition of AS-MWCNTs did not macroscopically alter the layer morphology of the printed TPU.

### 3.5. Mechanical Properties of AS-MWCNT/TPU FFF-Printed Parts

**Figure 5:**
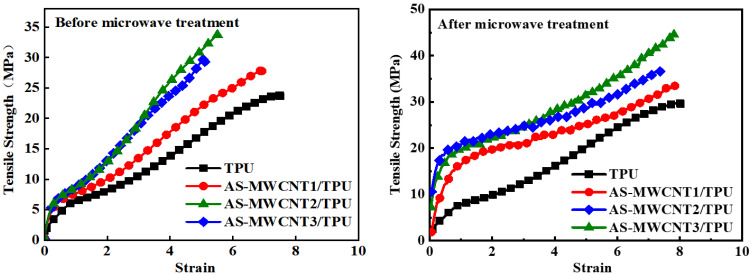
Comparative tensile stress–strain curves of AS-MWCNT/TPU FFF-printed parts before microwave treatment and after microwave treatment.

The tensile properties of FFF-printed TPU and AS-MWCNT/TPU composite samples were analyzed using a universal testing machine, with the results presented in Figure 5. It was observed that with increasing AS-MWCNT content, the tensile strength of the printed composite samples initially increased and then decreased, while the elongation at break showed a continuous decline. Among them, the printed AS-MWCNT2/TPU composite sample achieved a tensile strength of 34.8 MPa, representing a 43.8% increase compared to pure TPU (24.2 MPa). Meanwhile, its elongation at break decreased to 550%, which was 27.3% lower than that of pure TPU (757%). It can be seen that AS-MWCNTs exhibited significant reinforcement of TPU, as the amino-functionalized AS-MWCNTs—possessing superior mechanical properties—enhanced interfacial adhesion with the TPU matrix, thereby facilitating efficient load transfer and consequently improving tensile strength and modulus. Concurrently, the incorporation of rigid AS-MWCNTs restricted polymer chain mobility, leading to a concomitant reduction in elongation at break, with the extent of decrease correlating with filler loading. This behavior is consistent with established trends reported in the prior literature on CNT/polymer nanocomposite reinforcement mechanisms [36,37].

In the microwave electromagnetic field, the anisotropic polarization effect induced by the helical structure of AS-MWCNTs led to an asymmetric distribution of intramolecular charges, generating continuous dipole moment oscillations along the helical axis. This periodic reorientation of dipoles was strongly coupled with the alternating microwave electric field, converting electromagnetic energy into thermal energy via molecular friction. Therefore, in this study, AS-MWCNTs were introduced into the TPU material with the expectation that they would generate heat under microwave conditions, thereby welding the interlayer bonds of the AS-MWCNT/TPU FFF-printed parts. Accordingly, the tensile properties of microwave-treated TPU and AS-MWCNT/TPU FFF-printed parts were analyzed. It was found that after microwave treatment, the reinforcing effect of AS-MWCNTs on TPU became more pronounced. Specifically, the tensile strength of the AS-MWCNT2/TPU FFF-printed part reached 44.7 MPa, representing a 28.5% increase compared to the AS-MWCNT2/TPU specimen before microwave treatment. The tensile strength of the AS-MWCNT1/TPU FFF-printed part reached 33.7 MPa, marking a 21.6% improvement over its untreated counterpart. Furthermore, and more notably, the elongation at break of the AS-MWCNT/TPU FFF-printed parts after microwave treatment increased significantly. The elongation at break of the AS-MWCNT2/TPU FFF-printed part reached 785%, which was close to that of TPU (805%).

**Figure 6:**
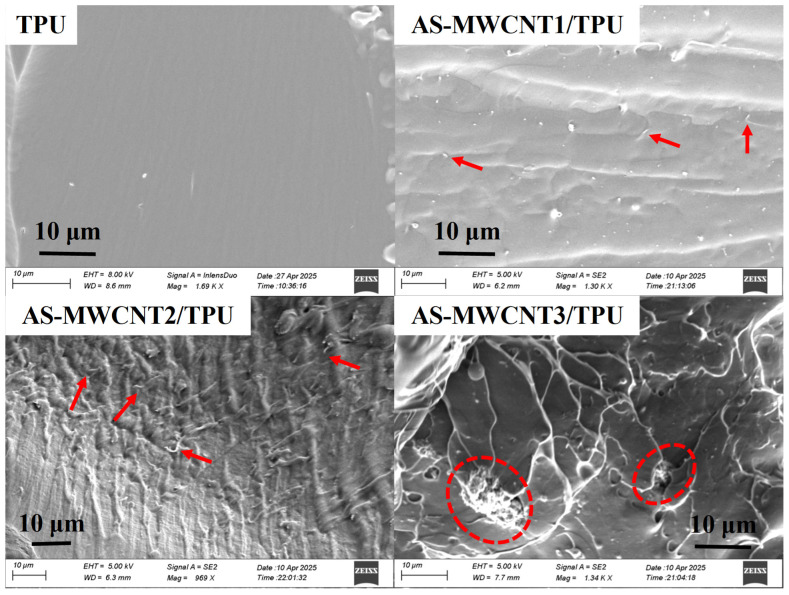
SEM images of fracture surfaces of TPU and AS-MWCNT/TPU FFF-printed parts.

To investigate the reinforcing mechanism of AS-MWCNTs in TPU, SEM analysis was conducted on the fracture surfaces of the printed TPU and AS-MWCNT/TPU composite samples, as shown in Figure 6. It was found that the fracture surface of pure TPU was smooth, whereas the fracture surfaces of the AS-MWCNT/TPU composite samples became progressively rougher with increasing filler content. In the printed AS-MWCNT1/TPU and AS-MWCNT2/TPU composite samples, pulled-out AS-MWCNTs, as the red arrows indicate, were visible, indicating good interfacial interaction between the AS-MWCNTs and the TPU matrix [38,39]. However, agglomerates of AS-MWCNTs were observed on the fracture surface of the AS-MWCNT3/TPU composite sample, suggesting that AS-MWCNT aggregation, as the red dashed circles indicate, occurred in this composite.

In summary, at 1–2 wt%, amino groups on AS-MWCNTs formed hydrogen bonds with urethane and ether segments in TPU, enabling uniform dispersion and a 3D interpenetrating network that enhanced stress transfer nanotubes acting as rigid load-bearing skeletons, suppressing microcrack initiation and increasing tensile strength; at 3 wt%, agglomeration disrupted dispersion, creating stress concentrators where modulus mismatch triggered interfacial debonding and rapid crack propagation along agglomerate edges, while excess nanotubes restricted chain mobility, hindering hard-segment rearrangement and soft-segment slippage, thereby weakening stress relief and causing tensile strength to peak at 2 wt% before declining due to competing reinforcement and failure mechanisms, with AS-MWCNTs persisting as physical cross-links that progressively reduced elongation at break by suppressing entropic recovery of soft segments, adsorbing chains to form rigid interfacial layers that lowered viscoelasticity and promoted brittle fracture, even as strain-induced crystallization transiently elevated modulus at the cost of ductility, and irreversible chain constraint sustained low elongation across all loadings.

**Figure 7:**
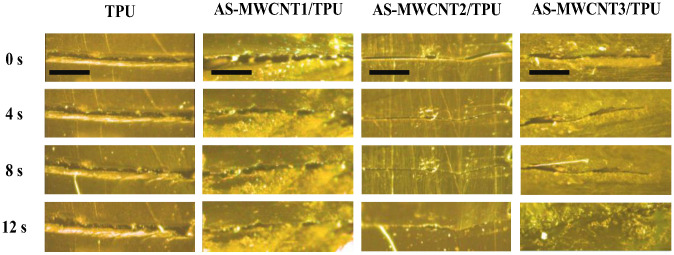
Morphologies of cracks in TPU and AS-MWCNT FFF-printed parts under varying microwave treatment durations (scale bar represented by black rectangle: 1 mm).

To track the healing process of interlayer defects in FFF-printed parts induced by microwave radiation, a deliberate scratch was introduced on all printed samples and subsequently exposed to microwave irradiation for varying durations, with healing progress observed as shown in Figure 7. For pure TPU FFF-printed parts, no noticeable change in the scratch was observed after 12 s of microwave exposure, indicating a lack of self-healing capability under this irradiation duration. In the case of AS-MWCNT1/TPU, partial healing occurred after 12 s, yet the overall scratch remained visible, suggesting that the healing effect was still insufficient at this stage. In contrast, AS-MWCNT2/TPU and AS-MWCNT3/TPU exhibited progressively improved healing with extended irradiation time. The sample with lower AS-MWCNT content showed only localized recovery, whereas the scratch on AS-MWCNT2/TPU was nearly fully healed. These comparative results clearly demonstrate the effectiveness of AS-MWCNT in enabling self-healing of FFF-printed parts under microwave irradiation.

**Figure 8:**
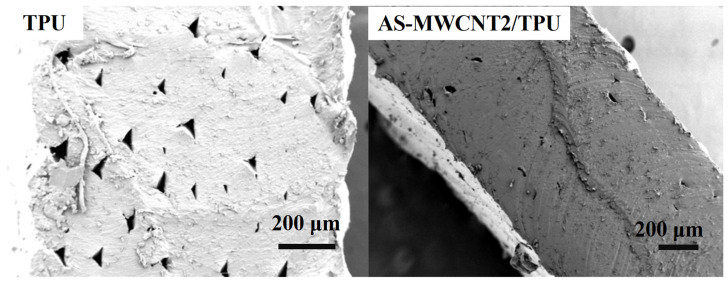
Cross-sectional SEM images of TPU and AS-MWCNT2/TPU FFF-printed parts after microwave treatment.

SEM observation was performed to analyze the structure of AS-MWCNT2/TPU FFF-printed parts after microwave treatment, as shown in Figure 8. It was observed that under microwave irradiation, the heat generated by the AS-MWCNTs acted as a welding agent, softening the TPU print layers. This enhanced the bonding between layers, leading to the reduction or elimination of defects inherent to the FFF process and resulting in a denser structure. This increased densification contributed to the improved tensile strength and elongation at break.

### 3.6. Influencing Factors and Condition Optimization for Supercritical Foaming of AS-MWCNTs/TPU FFF-Printed Parts

**Figure 9:**
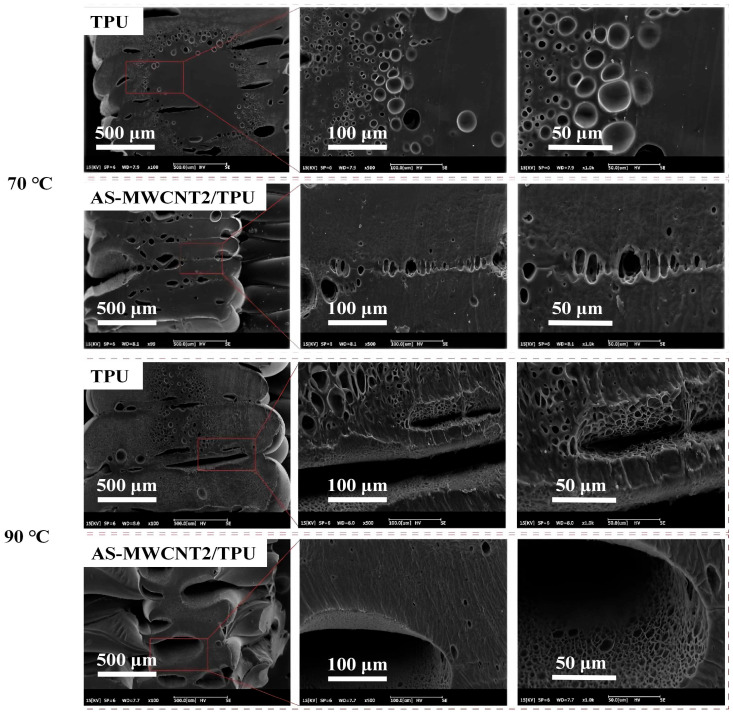
SEM images of TPU and AS-MWCNT2/TPU FFF-printed parts foamed at a foaming temperature of 70 °C and 90 °C.

The effects of different foaming temperatures on the foaming behavior of TPU and AS-MWCNT2/TPU FFF-printed parts after microwave treatment were investigated. For when the foaming temperature was 70 °C, the foaming results are shown in Figure 9. It can be observed that the AS-MWCNT2/TPU sample exhibited foam formation exclusively in the outer regions, while the interior remained unfoamed, indicating incomplete foaming and limited dissolved gas content within the polymer matrix. Similarly, the pristine TPU sample failed to develop a continuous pore structure, with only nascent pore nuclei observed. Pores were discernible at the gaps between printed filaments, demonstrating that at 70 °C, the high melt viscosity impeded effective CO_2_ penetration into the interior of the printed structure, thereby hindering nucleation [40]. Upon increasing the foaming temperature to 90 °C, distinct surface wrinkling was observed in both samples, accompanied by the formation of large-diameter pores on the surface, while internal pores remained relatively small. On one hand, when the foaming temperature exceeded the optimal expansion threshold, pore coalescence occurred, leading to post-expansion collapse and a broadening of the pore size distribution; at the surface and near-surface regions, where melt viscosity was lower, pores were more prone to aggregation and collapse. On the other hand, comparison between TPU and AS-MWCNT2/TPU reveals that the pristine TPU exhibited a significantly more heterogeneous pore size distribution, attributable to the incorporation of AS-MWCNT, which enhanced melt strength, reduced the nucleation energy barrier, and suppressed pore collapse and coalescence, thereby yielding a more uniform pore size distribution relative to pure TPU [23].

**Figure 10:**
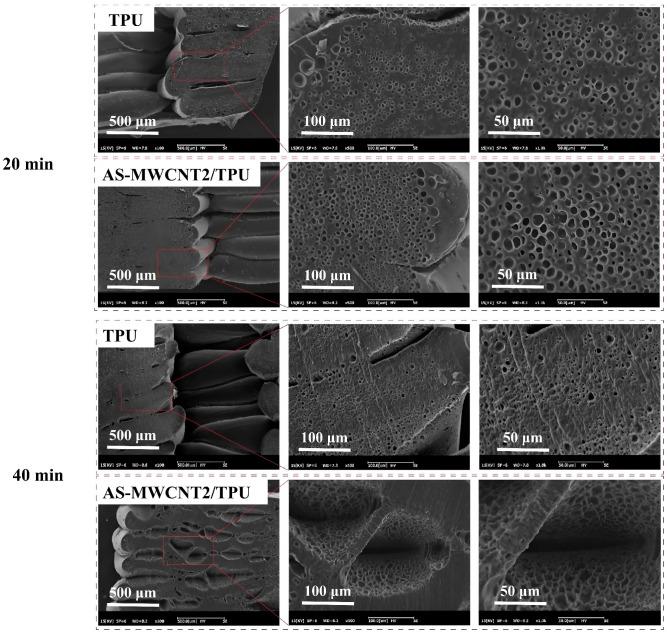
SEM images of TPU and AS-MWCNT2/TPU FFF-printed parts foamed at a gas saturation time of 20 min and 40 min.

The effects of different saturation times on the foaming behavior of TPU and AS-MWCNT2/TPU FFF-printed parts were investigated. For when the saturation time was 20 min, the foaming results are shown in Figure 10. As observed, both the TPU and AS-MWCNT2/TPU FFF-printed parts developed only pores with relatively small diameters, indicating incomplete foaming. This suggests that the gas saturation time was insufficient for CO_2_ to fully diffuse and dissolve within the polymer matrix. When the saturation time was increased to 40 min, it can be seen that the surfaces of both the TPU and AS-MWCNT2/TPU FFF-printed parts exhibited significant wrinkling. This indicates that the saturation time was excessive, leading to over-expansion followed by substantial shrinkage of the foam structure. These results demonstrate that the pore size and foam morphology can be regulated by adjusting the saturation conditions.

**Figure 11:**
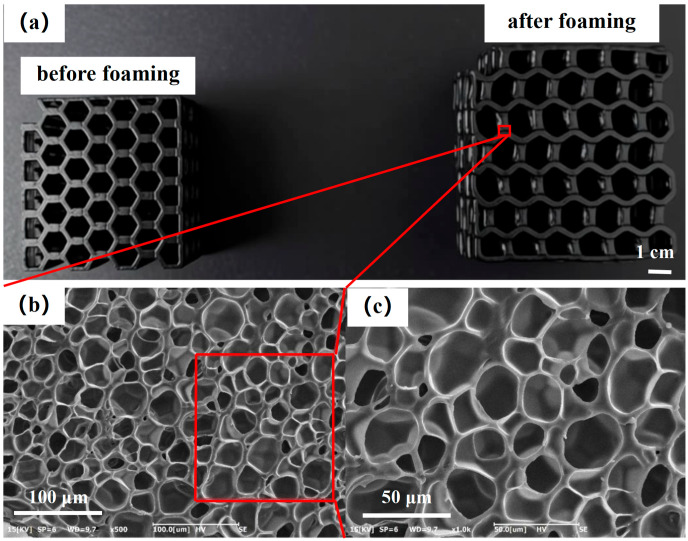
(**a**) AS-MWCNT2/TPU FFF-printed parts before and after foaming and (**b**,**c**) SEM images of foamed AS-MWCNT2/TPU FFF-printed parts.

Based on the results of different foaming temperatures and gas saturation times, the optimal foaming conditions for TPU and AS-MWCNT2/TPU FFF-printed parts were 80 °C for 30 s, with a gas saturation time of 30 min. Under these parameters, AS-MWCNT2/TPU FFF-printed parts were foamed, and their cell morphology was examined (Figure 11). The resulting foam exhibited a uniform cell structure with well-preserved cell integrity.

## 4. Conclusions

In summary, this work proposed a microwave-assisted self-healing FFF-printed polymeric material and its supercritical foaming. AS-MWCNTs were compounded with TPU and melt-extruded to produce printable filaments. These filaments were then processed via FFF, followed by microwave irradiation to yield TPU FFF-printed parts with enhanced interlayer performance. AS-MWCNTs not only improved interfacial interactions with TPU through amination and their unique helical geometry, thereby increasing tensile strength and modulus, but also possessed intrinsic chiral parameters that enabled cross-polarization under electromagnetic fields, granting exceptional microwave absorption capacity. Upon microwave irradiation, the nanotubes rapidly converted electromagnetic energy into localized heat, softening the surrounding TPU and enabling autonomous repair of interlayer defects inherent to FFF fabrication. This mechanism significantly enhanced the mechanical integrity and functional performance of thermoplastic FFF-printed components. The conditions of foaming temperature and gas saturation time were 80 °C and 30 s for supercritical foaming, and the resulting foam exhibited a uniform cell structure with well-preserved cell integrity.

## Data Availability

The original contributions presented in this study are included in the article/Supplementary Materials. Further inquiries can be directed to the corresponding author.

## Supplementary Materials

The following supporting information can be downloaded at https://www.mdpi.com/article/10.3390/nano16060384/s1: Figure S1: SEM Images of S-MWCNT and AS-MWCNT; Table S1: The selected mechanical results of TPU and AS-MWCNT/TPU FFF-printed parts before microwave treatment and after microwave treatment.
