# Supplementary material for: A Microwave-Assisted, Rapidly Self-Healing, FFF-Printed TPU and Its Application in Supercritical Foaming
Source: Nanomaterials (Basel). 2026 Mar 23;16(6):384. doi: 10.3390/nano16060384 (PMC13029445; doi:10.3390/nano16060384)
Supplement: Supplementary file 1 [file nanomaterials-16-00384-s001.zip › nanomaterials-4176269-supplementary.pdf]

A Microwave-Assisted, Rapidly Self-Healing, FFF-Printed TPU and Its Application in Supercritical Foaming

Shaoyun Chen [1,2,3], Rui Wang [1,2,3,*], Longhui Zheng [4], Jianhong Gao [1,2,3], Cuifang Cai [1,2,3], Zixiang Weng [4], Xiaoying Liu [1,2,3], Bo Qu [1,2,3], Jianlei Wang [4,*] and Dongxian Zhuo [1,2,3,*]

1 School of Chemical Engineering and Materials Science, Quanzhou Normal University, Quanzhou 360002, China

2 Fujian University Engineering Research Center of Polymer Functional Coating Based Graphene, Quanzhou 360002, China

3 Fujian Key Laboratory of New Materials for Light Textile and Chemical Industry, Quanzhou 360002, China

4 Fujian Key Laboratory of Nanomaterials, Fujian Institute of Research on the Structure of Matter, Chinese Academy of Sciences, Fuzhou 350002, China

* Correspondence: wangrui@fjirsm.ac.cn (R.W.); jlwang@fjirsm.ac.cn (J.W.); dxzhuo@qztc.edu.cn (D.Z.)

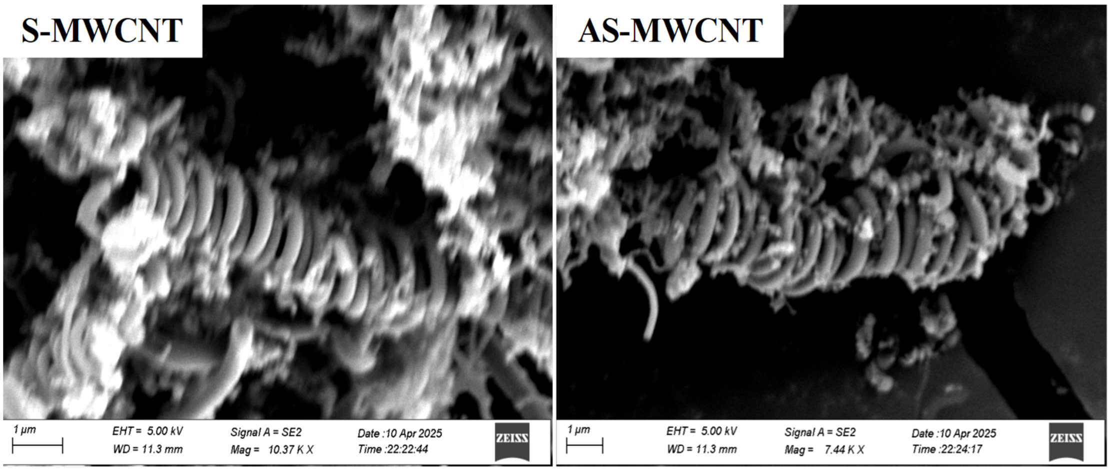

Figure S1. SEM Images of S-MWCNT and AS-MWCNT.

Table S1. The selected mechanical result of TPU and AS-MWCNT/TPU FFF-printed parts before microwave treatment and after microwave treatment

Samples	Before microwave treatment		After microwave treatment	
	Tensile strength (MPa)	Elongation at break (%)	Tensile strength (MPa)	Elongation at break (%)
TPU	24.2±0.3	757±34	27.8±0.3	802±31
AS-MWCNT1/TPU	27.5±0.5	687±46	33.7±0.4	790±37
AS-MWCNT2/TPU	34.8±0.5	550±36	44.7±0.3	785±34
AS-MWCNT3/TPU	28.9±0.8	507±56	34.6±0.5	742±54
